# Proximal Risk for Suicide: Protocol for an Ecological Momentary Assessment Study

**DOI:** 10.2196/37583

**Published:** 2022-07-12

**Authors:** Pravesh Sharma, Robert Peck, Anthony R Sinicrope, Thomas Pavey, Jennifer J Muehlenkamp

**Affiliations:** 1 Department of Psychiatry and Psychology Mayo Clinic Health Systems Eau Claire, WI United States; 2 Behavioral Health Reseach Program Mayo Clinic Rochester, MN United States; 3 Department of Psychology University of Wisconsin Eau Claire Eau Claire, WI United States

**Keywords:** suicide, suicide risk, suicide ideation, suicide prevention, diary, ecological momentary assessment, Integrative-Volitional-Motivational Theory

## Abstract

**Background:**

Suicide is a prevalent public health concern in the United States across all age groups. Research has emphasized the need to identify risk markers that prevent suicide along shorter timeframes, such as days to weeks. Furthermore, little has been done to explore the relative significance of factors that can predict short-term suicide risk or to evaluate how daily variability in these factors impacts suicidal ideation or behavior. This proposed project aims to identify risk factors that best predict near-time changes in suicidal ideation and examine potential interactions between these factors to predict transitions into suicidal thinking or behaviors.

**Objective:**

The aim of this proposed study is threefold: (1) To identify which psychological risk factors are most strongly associated with proximal changes in suicide risk across days and weeks. (2) To evaluate theoretical assumptions of the Integrative-Motivational-Volitional Theory of Suicide. (3) To determine how disruptions in physiological arousal interact with theoretical mechanisms of risk to predict concurrent and short-term prospective increase in suicidal thoughts and behaviors.

**Methods:**

A daily diary or ecological momentary assessment design will be utilized with 200 participants. Participants will complete 2 in-person visits separated by 3 weeks during which they will complete 3 brief daily assessments within their natural environments using the ilumivu research app on a smart device. Research will occur at the Mayo Clinic Health System (MCHS) Eau Claire site. Participants will be recruited through chart review and standard care delivery assessment.

**Results:**

This manuscript outlines the protocol that will guide the conduct of the forthcoming study.

**Conclusions:**

The proposed project aims to lead efforts using technological advances to capture microchanges in suicidal thinking/behavior over shorter timeframes and thereby guide future clinical assessment and management of suicidal patients. Results of this study will generate robust evidence to evaluate which risk factors predict proximal changes in suicidal ideation and behaviors. They will also provide the ability to examine potential interactions with multiple theoretically derived risk factors to predict proximal transitions into worsening suicidal thinking or behaviors. Such information will provide new targets for intervention that could ultimately reduce suicide-related morbidity and mortality.

**International Registered Report Identifier (IRRID):**

PRR1-10.2196/37583

## Introduction

### Background

Deaths from suicide continue to rise within the United States across all age groups [1]. Despite it being a significant public health concern, little progress has been made over the past 50 years in our ability to differentiate those at risk of suicidal thoughts versus attempts [2]. While many risk factors, warning signs, and predictors have been identified for suicidal risk broadly, most research has focused on factors that predict suicide within the next year [2]. This is of minimal utility for the clinician trying to evaluate whether a patient may attempt suicide in the coming hours and days. Researchers emphasize the need to identify risk markers that predict suicide in days to weeks [3,4], but this work remains scarce. Even within this inquiry into shorter-term risk, most studies focus on single psychiatric or demographic risk factors, which is problematic given that suicide is a complex phenomenon involving multiple interacting factors [5,6]. Major advances in the prevention of suicide require studies that examine interacting processes that covary with and immediately precede changes in suicidal behavior [2,7].

Some of the factors that appear to predict short-term risk for escalation in suicidal thinking or attempts include specific hopelessness about life concerns [8,9], engagement in nonsuicidal self-injury (NSSI; [10]), interpersonal stressors including perceived burdensomeness and lack of belonging [11], substance use [2], poor sleep quality/insomnia [12,13], agitation [14], and self-criticism/self-hate [15]. However, only few of these factors have been examined simultaneously to determine the most important ones to discerning suicide risk; besides, none have been studied to evaluate how daily variability in these factors impacts daily changes in suicidal thinking or behavior. This study begins to address these scientific and clinical questions by identifying which of the noted factors hold the strongest association with short-term suicide-related crisis escalation within and across days, in addition to examining potential interactions among the variables.

Recent theoretical models of suicide acknowledge the volatile nature of suicidal thinking/risk across hours and days, stressing the importance of identifying how changes in theorized risk factors interact to facilitate transitions from suicidal thinking to suicidal actions. The Integrative-Motivational-Volitional (IMV) Theory of Suicide [16] specifies that transitions to suicidal thinking and behavior are determined by different state-specific processes that interact with preexisting states of distress (eg, entrapment, hopelessness) to either facilitate or hinder movement between thinking and acting. Motivational factors (eg, loneliness, burdensomeness, specific hopelessness) are theorized to impact the development of suicidal ideation, whereas volitional factors (eg, self-hate, agitation, reduced fears of death, engagement in NSSI) impact transition to suicidal behavior. While there is support for the IMV, almost all the research has been cross sectional, exploring single motivational moderators impacting the emergence of suicidal ideation (eg, [17,18]). To date, there are only 2 known studies [19,20] that have evaluated the daily or weekly relationship between motivational or volitional factors and the transition to suicidal behavior. One study evaluated the motivational factors of hopelessness, thwarted belonging, and perceived belongingness within 74 patients who completed a 6-day ecological momentary assessment (EMA) protocol [20]. They found that hopelessness and perceived burdensomeness prospectively predicted both passive and active suicidal ideation, and the interaction between thwarted belonging and perceived burdensomeness also predicted active ideation. These results were similar to those reported by Al-Dajani and Czyz [19] in a sample of 78 adolescents hospitalized for suicide risk. The limited research examining proximal changes in motivational and volitional factors for suicide ideation and behavior represents a major gap in the field and is essential to evaluating the clinical validity of the IMV theory. In addition, studies have yet to explore how motivational and volitional factors interact among themselves to influence risk escalation and how other psychological processes (eg, sleep quality) may impact these factors and their temporal variability in risk transition. The proposed project will be the first to provide data testing these theoretical assumptions.

Recent studies of acute risk for suicide have identified that forms of physiological arousal such as agitation [21] and sleep difficulties [22] are linked to risk for suicidal behavior [23]. Meta-analyses consistently identify that insomnia, disrupted sleep patterns, and especially nightmares are linked to increased suicide risk [12,13,22,24]. Studies evaluating interactions between variability in sleep disturbances and other psychological processes (eg, agitation, self-hate, burdensomeness) believed to impact suicide risk are lacking. This study will advance scientific knowledge and potentially improve the utility of suicide theories, such as the IMV for clinical practice, by examining how sleep disturbances impact psychological processes, such as agitation, to predict near-term changes in suicidal thinking or behaviors.

We know that suicidal ideation and behavior are fluid across hours and days, and that suicidal crises are typically brief [3,25,26]. Most of the existing research relies on long-term retrospective recall; little research uses prospective designs and many use hospital records, which provide limited details of individual experiences. EMA studies have participants respond to brief surveys multiple times daily, providing an avenue to identify acute precursors of problematic behavior and contribute new insights into the factors impacting the fluid nature of suicidal ideation and behavior. Assessing components of ideation-to-action transitions through an EMA methodology has the advantage of relying on recall from the past hours rather than recall over days, weeks, and months. Numerous experts have called for an increase in the use of micro-longitudinal methods such as EMA to study suicide [27,28], yet these studies remain rare [29].

To date, there are a few known studies that have utilized EMA research designs to obtain data about the within- and between-day changes in suicidal thinking (our study will be among the first to also examine suicidal behaviors). The first study was conducted by Kleiman and colleagues [26]. They enrolled 90 adults with a recent suicide attempt recruited from the social media platform Reddit or inpatients hospitalized for suicide attempts. These participants completed up to 28 days of EMA data collection that included 4 randomly signaled assessment prompts separated by 4-8 hours. Results revealed that suicidal ideation severity fluctuated markedly across hours, as did the risk factors assessed. The variability in the risk factors experienced was significantly correlated with concurrent changes in suicidal thinking. Three similar studies, all conducted with adolescents discharged from acute psychiatric care [30-32], found similar results in that suicidal ideation significantly fluctuated across hours and covaried with similar fluctuations in assessed risk factors. Relevant to the current proposal, a recent EMA study (1-week duration; 6 assessment prompts/day) of 51 adults with current suicidal ideation found that a shorter sleep duration predicted higher severity of suicidal ideation the next day. Furthermore, Hallensleben and colleagues [20] reported that both passive and active ideation demonstrated significant within- and moment-to-moment variability, which was associated with momentary variability in hopelessness and perceived burdensomeness. These studies provide some of the first and much needed, fine-grained data to better understand how suicide risk varies across short intervals. They provide proof of concept and feasibility of using EMA to study suicide safely but remain primarily descriptive and fail to test prominent theoretical assumptions detailing transitions to increased suicidal thinking and behaviors.

This proposed project will be one of the first to better define which risk factors predict near-time changes in suicidal ideation and provide data on potential interactions among multiple theoretically derived risk factors to predict proximal transitions into worsening suicidal thinking or behaviors. The ability to provide data capable of identifying proximal indicators of risk and temporal sequencing of risk factors to suicidal behaviors addresses a suicide research priority [29,33], and the data to be collected will also enable a variety of additional exploratory aims related to the IMV theory to be evaluated. To truly advance the field of suicide research and prevention, researchers must begin to use innovative methodologies, such as EMA, to assess patients at frequent intervals to understand the acute evolution of suicidal crises. This study will fill a gap in the existing research on suicide, providing a fine-grain assessment of theoretical risk factors believed to impact the processes that influence suicidal crisis and provide clinicians with more ecologically valid risk indicators to monitor with their patients.

### Study Aims and Objectives

The purpose of this study is to better understand suicide risk among patients by identifying the daily markers/factors associated with near-term (proximal) escalation in suicide thinking and behaviors among patients so that clinicians can be better prepared to monitor risk and intervene.

The aims of this study are as follows:

1. To identify which psychological risk factors are most strongly associated with proximal changes in suicide risk across days and weeks to enhance clinical risk monitoring.
  - Hypothesis 1a: Based on the IMV theory predictions [16] as well as recently published work (eg, [19,20]), within-day variability of hopelessness, self-hate, agitation, entrapment, burdensomeness, and connectedness will be associated with same-day moment-to-moment variability in suicidal ideation and behaviors.
  - Hypothesis 1b: Participants will be more likely to report increases in suicidal ideation on days when they experience increases in hopelessness, burdensomeness, entrapment, and decreases in connectedness (see [19,34]).
  - Hypothesis 1c: Given recent work demonstrating that poor sleep quality predicted next-day increases in suicidal ideation (eg, [35]), we also hypothesize that participants will be more likely to report engagement in suicidal behaviors on days when sleep quality is poor, and self-hate and agitation are high.
2. To evaluate theoretical assumptions of the IMV [16] theory describing transitional processes from suicidal ideation to behavior:
  - Hypothesis 2a: According to the IMV theoretical predictions and cross-sectional studies (eg, [17]), we hypothesize that changes in entrapment and hopelessness will produce changes in suicidal ideation and the strength of the association will be moderated by variability in burdensomeness, connectedness, and self-hate (eg, motivational moderators).
  - Hypothesis 2b: Changes in suicidal ideation will co-occur with changes in suicidal behavior and the strength of the association will be moderated by variability in NSSI engagement/urges, self-hate, and agitation (eg, volitional moderators; [10,16]).
3. To determine how disruptions in physiological arousal (sleep problems/substance use) interact with theoretical mechanisms of risk [16] to predict concurrent and short-term prospective increases in suicidal thoughts and behaviors.
  Drawing from empirical studies showing that disruptions in sleep (eg, [35,36]) and increases in substance abuse (eg, [37]) are related to increased suicidal thoughts and behaviors, we hypothesize that:
  - Hypothesis 3a: Daily (weekly) changes in sleep quality (and substance use) will be associated with next day (week) changes in hopelessness, self-hate, burdensomeness, entrapment, and agitation to predict changes in suicidal ideation and behaviors (also see [38]).
  - Hypothesis 3b: Daily (weekly) variability in sleep quality (substance use) will interact with entrapment, hopelessness, and the motivational moderators of connectedness and burdensomeness to predict concurrent and next day (week) increases in suicidal ideation.
  - Hypothesis 3c: Daily (weekly) variability in sleep quality (substance use) will interact with suicidal ideation, self-hate, and agitation to predict concurrent and next-day (week) engagement in suicidal behaviors.

## Methods

### Study Design

This study uses a micro-longitudinal study design, also known as a daily diary or EMA. We will use EMA to refer to this study design throughout this proposal. The general study procedure includes participants completing 2 in-person visits, separated by 3 weeks, during which they will complete 3 brief (approximately 5 minutes) daily assessments within their natural environments through the ilumivu research app downloaded onto their smartphone or a similar device (total daily time commitment required is approximately 15 minutes). The first in-person visit will be used to enroll participants into the study and begin active data collection, including training participants to use the EMA study app. After the 3 weeks, participants will return for a final in-person visit to conclude their participation.

### Recruitment

This research will occur at the Mayo Clinic Health System Eau Claire site. Study staff will identify potential participants through chart review and standard care delivery assessment processes. Patients who meet inclusion criteria for suicidal thinking (ie, a score of ≥1 in item 9 of the Patient Health Questionnaire or a “yes” response to item 2 in the Columbia-Suicide Severity Rating Scale [C-SSRS] screening tool and are patients involved with behavioral health services) will be recruited for the study. Eligible patients will be contacted through their patient portal with information about the study and invited to participate in the study by reaching out to study staff via email or phone. Treating clinicians may also identify potential participants who will briefly inform the patient about the study, stating that the study staff will contact them if the patient expresses interest in participating. The referring clinician would then provide the patient’s name to the research study coordinator. Study staff will contact potential participants, provide additional study information, and schedule a 1:1 meeting to obtain consent/collect baseline data.

### Baseline Data Collection Visit (Visit 1)

**Figure 1:**
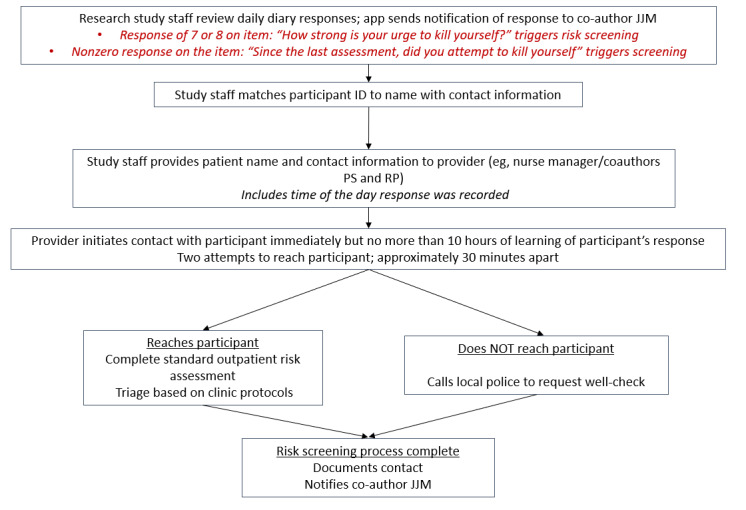
Safety screening process flowchart.

Study staff will meet with a potential participant in a private room within the outpatient behavioral health clinic to gather participant consent to participate in the study. Study staff will verbally review the informed consent form with participants, including a careful review of the study safety protocol (Figure 1), answering questions and concerns. Patients agreeing to participate will sign the informed consent form and be given a copy for their records.

Following the consenting process, participants will complete the baseline data collection protocol consisting of self-report questionnaires and behavioral reaction time tasks on an iPad provided by the study staff. The baseline questionnaires will be accessed through a hyperlink to a study-specific survey housed on Inquisit, a secure online survey administration tool. The baseline assessments include psychometrically validated comprehensive measures of the primary variables of interest to the study that will be presented in a random order across participants to reduce ordering effects on the data quality. The content of the baseline assessment can be seen in Multimedia Appendix 1. Following baseline data collection, participants will be introduced to the ilumivu EMA app. Study staff will assist participants with downloading the required apps onto their smartphone device. Participants will be trained on using the EMA app for data collection and complete their first EMA entry.

**Figure 2:**
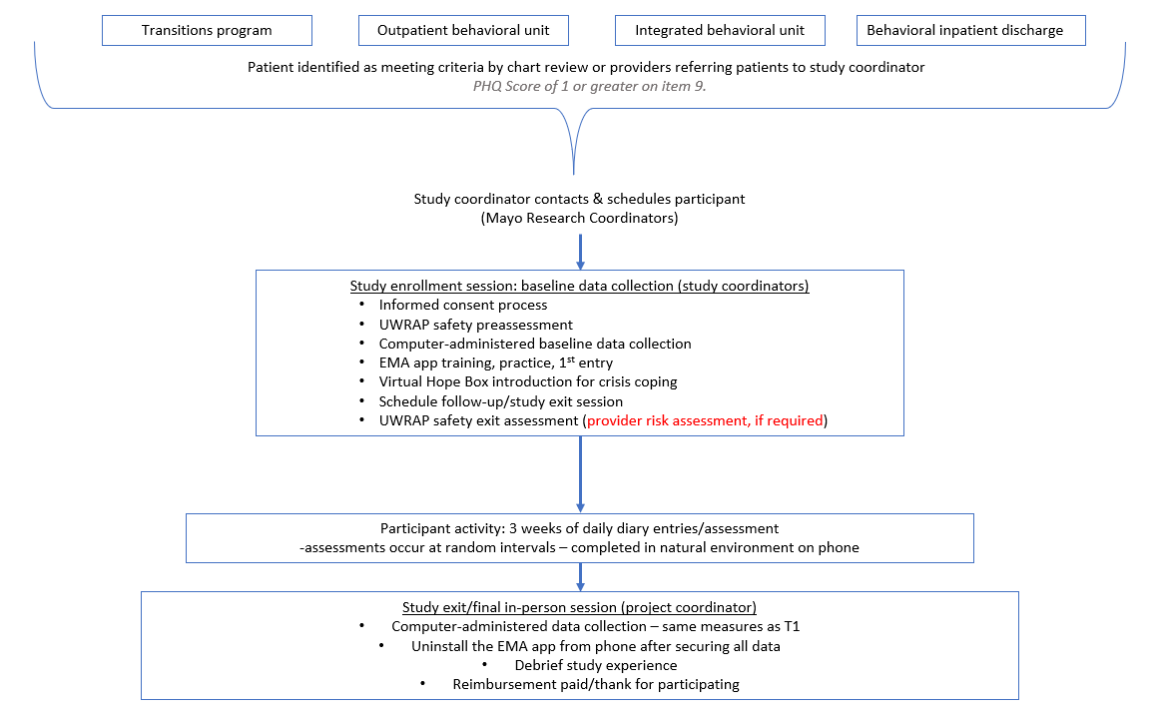
Study process workflow. EMA: ecological momentary assessment; PHQ: Patient Health Questionnaire; UWRAP: University of Washington Risk Assessment Protocol.

After completing their first EMA data entry on their smartphone, participants will be introduced to the Virtual Hope Box app, which is a freely accessible app that has demonstrated effectiveness as a tool to manage and cope with suicidal urges [39,40]. Participants will be encouraged to download this app to help manage future suicidal impulses as part of the study safety protocol, in addition to being provided local and national crisis response resources. At the end of this session, participants will be compensated for their initial visit (US $30.00) and schedule their final session visit appointment (Figure 2).

### Three-Week EMA Data Collection

Starting on the day of the study enrollment, participants will begin the EMA data collection phase of the study. On each day for a 3-week period, participants will be notified to log into the EMA app on their smartphone 3 times throughout the day (7-10 AM, 1-4 PM, and 6-9 PM). Each assessment will include single-item questions evaluating the study variables and should take approximately 5 minutes to complete. Questions about sleep quality will only be asked during the first daily assessment and questions about substance use and medication adherence will only be asked during the third/final assessment of each day. The content for each daily diary assessment can be accessed through Multimedia Appendix 2. Participants who have not responded during a survey window will receive a push notification to remind them to complete their survey before it closes (within 30 minutes of the timeframe).

### Study Completion (Final Visit)

Study staff will contact participants before their scheduled study completion visit to confirm the appointment or reschedule if necessary. At this visit, participants will complete the same set of computer-administered questionnaires that were completed during the enrollment visit, allowing for longitudinal data analysis with comprehensive measures of the study variables. Study staff will ensure participants safely uninstall the ilumivu EMA software app from their smartphones and will debrief the study experience. Questions will be answered, and participants will receive their prorated compensation based on visits/daily assessments completed.

### Analysis

Research aim 1 focuses on testing the coupled associations between within-day variability of psychological risk factors and within-day variability of suicidal thoughts and behaviors. For Hypothesis 1a, the root mean square of successive differences (RMSSDs) can be used to quantify within-person variability in clinical indicators while accounting for temporal dependency, amplitude, and frequency [41]. We will calculate the within-day RMSSD of each psychological risk factor (ie, hopelessness, self-hate, agitation, entrapment, burdensomeness, and connectedness) and each outcome (ie, suicidal ideation and suicidal behaviors). The RMSSD of each risk factor will then be used as a level 1 predictor of same-day RMSSD of each outcome in a multilevel linear model. We will include any demographic covariates in the level 2 model. A lagged model will assess whether variability in risk factors on day *t* predicts suicide outcomes on day *t* + 1, controlling for variability in risk factor at *t* + 1. To test hypothesis 1b, we will examine the within-day coupled association between the average level of suicidal ideation in each assessment and the average level of each psychological risk factor (ie, hopelessness, self-hate, agitation, entrapment, burdensomeness, and connectedness). Risk factors will be entered as level 1 predictors in separate multivariate linear models (MLMs), with suicidal ideation as the dependent variable. To test hypothesis 1c, we will first create a daily average of self-hate and agitation (each measured 3 times per day) using the mean score of available reports. We will then run separate MLMs with sleep quality, average self-hate, and average agitation as level 1 predictors of daily suicidal behavior. As noted earlier, we expect that suicidal behavior will be infrequent even in this clinical sample. Thus, we anticipate that zero-inflated or binomial models may fit the data better than a linear model.

Research aim 2 focuses on testing moderators of the coupled relationships tested in aim 1 and hypotheses 2 and 3. We will again use multilevel linear models to test these relationships. To test hypothesis 2a, we will manually calculate the interaction between each person-centered focal predictor (ie, entrapment, hopelessness) and each person-centered moderator (ie, burdensomeness, connectedness, and self-hate). Each combination of focal predictor, moderator, and interaction will be entered as level 1 effects in an MLM predicting suicidal ideation. To test hypothesis 2b, we will again manually calculate the interactions between the person-centered focal predictor (ie, suicidal ideation) and moderators (ie, NSSI urge/engagement, self-hate, and agitation), and then enter combinations of each into separate MLMs predicting suicidal behavior.

Research aim 3 focuses on examining psychological risk factors as potential mediators of the relationship between physiological arousal (ie, sleep problems, substance use) and suicidal ideation or behavior. Unlike the previous contemporaneous models, which focus on within-day effects, these models also include lagged effects, with predictors on day *j* predicting changes in the outcome on day *j*+1. To account for these additional complexities, we will use multilevel structural equation modeling, which offers a number of advantages when testing multilevel mediation (see [42]), including an autoregressive mediation model appropriate to these hypotheses.

To test hypothesis 3a, each model will include direct paths between one of the focal predictors (ie, daily sleep or substance use) and next-day suicidal ideation or behavior as well as indirect paths through each of the 5 hypothesized mediators (ie, daily averages of hopelessness, self-hate, burdensomeness, entrapment, and agitation) lagged by 1 period (ie, *j*+1), controlling for the autocorrelations of each predictor on days *j* and *j*+1.

To test hypothesis 3b, we will examine (1) the main effect of variability (ie, RMSSD) in daily sleep quality; (2) the main effects of daily entrapment, hopelessness, and burdensomeness; and (3) the interactions of (1) and (2) in predicting same-day suicidal ideation. We will then extend this model to predict next-day suicidal ideation, controlling for the autocorrelation of suicidal ideation on the previous day. Similarly, to test hypothesis 3.3, we will examine whether variability in sleep quality interacts with average daily suicidal ideation, self-hate, or agitation to predict same-day or next-day (lagged) suicidal behavior. These models will again use standard MLM, with level 1 interactions manually computed from the person-centered variables and entered as predictors along with the main effects of each focal predictor and moderator.

### Power Statement

To determine our planned sample size, we conducted power analyses using the ema.pcurve function of the EMAtools package [43]. With 200 participants responding to as few as 75% of survey requests, we will obtain 47.25 data points per participant (200 × 47.25 responses = 9450 datapoints), which is sufficient to detect medium to large effects (Cohen *d* > 0.5) at over 90% power and small effects (Cohen *d* > 0.2) with at least 30% power. This sample will be larger than many of the existing suicide-focused EMA studies to date (eg, [20,26]). Thus, the sample size is adequate for a preliminary study.

### Ethics Approval

The study was approved by the Mayo Clinic Institutional Review Board (ID: 20-005229) after obtaining and implementing expert peer review comments from the Psychiatry and Psychology Research Committee.

## Results

While the methodologies used to conduct this study pose minimal risk to participants, this study does include self-report questions about suicidal thoughts and is recruiting participants with some level of suicidal thinking; therefore, it requires participant safety monitoring. This study will utilize the Research Protocols and Risk of Suicide guidelines [44] published by the UCLA (University of California at Los Angeles) Office of the Human Research Protection Program (2012), as recommended by the National Institutes of Health (NIH), to ensure the safety of participants as well as the expert consensus recommendations for conducting EMA studies of suicide [45]. The guidelines note that studies that identify participants who may have current suicidal ideation through direct questions about suicidal thoughts and plans, or through direct questions regarding known risk factors for suicide need a patient safety monitoring system in place. Based on the guidelines, we will have multiple risk management procedures in place (Figure 1).

## Discussion

This project will generate robust evidence to evaluate which risk factors predict proximal changes in suicidal ideation and behaviors. It also provides an innovative ability to examine potential interactions with multiple theoretically derived risk factors to predict proximal transitions into worsening suicidal thinking or behaviors. In sum, the study has practical and clinical implications and will inform clinicians of a better understanding of the real-world, daily events that can lead to escalations in suicidal crises, and is expected to contribute to advancements in clinical care of suicidal patients. In addition, the project has the potential to theoretically enhance the understanding of the elements that elevate suicide risk and will demonstrate the feasibility of app-based real-time monitoring of suicide risk factors that will have a huge impact on advancing innovative suicide prevention and treatment.

This proposed project will join and help lead a paradigm shift in the scientific study of suicide using technological advances (ie, EMA) to capture microchanges in psychological processes to predict acute changes in suicidal thinking and behavior. The information obtained from this study has excellent potential to guide clinical assessment and care of suicidal patients.

## Appendix

Multimedia Appendix 1 Baseline data collection visit.

Multimedia Appendix 2 Ecological momentary assessment data collection.

## Abbreviations

C-SSRS: Columbia-Suicide Severity Rating Scale
EMA: ecological momentary assessment
IMV: Integrative-Motivational-Volitional
MLM: multivariate linear model
NIH: National Institutes of Health
NSSI: nonsuicidal self-injury
RMSSD: root mean square of successive difference
UCLA: University of California at Los Angeles

## Data Availability

The data sets that will be generated during or analyzed during the study can be obtained from the corresponding author on reasonable request.
