# Supplementary material for: Proximal Risk for Suicide: Protocol for an Ecological Momentary Assessment Study
Source: JMIR Res Protoc. 2022 Jul 12;11(7):e37583. doi: 10.2196/37583 (PMC9328781; doi:10.2196/37583)
Supplement: Multimedia Appendix 1 [file resprot_v11i7e37583_app1.docx]

## **Multimedia Appendix 1. Baseline Data Collection Visit (Visit 1)**

The full baseline assessment protocol includes 193 items and 3 short behavioral reaction time tasks. It is expected to take participants approximately 60 – 90 minutes to complete the protocol:

### **Features of Suicidal Thoughts & Behavior: 190 items**

- Self-Injurious Thoughts and Behavior Inventory-Short form (SITBI; Nock et al., 2007; 16 items)
- Suicide Status Form (SSF; Jobes, 2016; 8 items)
- Death/Suicide Implicit Association Test (d/s IAT; Nock et al., 2010; reaction time task ~5min)
- Self-Harm Affect Misattribution Paradigm (SH-AMP; Franklin et al., 2014; reaction time task ~5min)

### **Constructs of Hopelessness: 22 items**

- Short Defeat & Entrapment Scale (SDES; Griffiths et al., 2015; 8 items)
- Interpersonal Hopelessness Scale (IHS; Tucker et al., 2018; 10 items)
- Brief Beck Hopelessness Scale (B-BHS; Forintos et al., 2013; 4 items)

### **Non-Suicidal Self-Injury: 46 items**

- NSSI Disorder Scale (NSSIDS; Victor et al., 2017; 20 items)
- Inventory of Statements about Self-Injury Short Form (ISAS-SF; Victor et al., 2016; 26 items)

### **Substance Use: 12 items**

- National Institute of Drug Abuse Quick Screen + Other Drugs (NIDA; NIDA 2011; 12 items)

### **Risk Factors: 55 items**

- Acquired Capability with Rehearsal Scale (ACWRSS; George et al., 2016; 7 items)
- Insomnia Severity Index (ISI; Bastien et al., 2001; 5 items)
- Self-Rating Scale (SRS; Hooley et al., 2010; 8 items)
- Short UPPS-P Impulsive Behavior Scale (Cyders et al., 2014; 8 items)
- Ruminative Response Scale – Brooding (RRS-B; Treynor et al., 2003; 5 items)
- Brief Agitation Measure (BAM; Ribeiro et al., 2011; 3 items)
- UCLA 3-item Loneliness Scale (UCLA; Russell, 1996; 3 items)
- Self-Criticism AMP (Fox et al., 2019; reaction time task ~5min)
- Depression/Anxiety Symptoms PHQ-4 (Kroenke et al., 2001; Spitzer et al., 2006; 4items)

### **Protective Factors: 52 items**

- Cognitive Emotion Regulation Questionnaire-short (CERQ-S; Garnefski et al., 2006; 18 items)
- Brief Reasons for Living Inventory (BRFL; Ivanoff et al., 1994; 12 items)
- Body Regard Scale-Brief (BRS; Muehlenkamp, 2012; 12 items)
