# Supplementary material for: Proximal Risk for Suicide: Protocol for an Ecological Momentary Assessment Study
Source: JMIR Res Protoc. 2022 Jul 12;11(7):e37583. doi: 10.2196/37583 (PMC9328781; doi:10.2196/37583)

## **Multimedia Appendix 2. Three-week EMA Data Collection: EMA Daily Assessment Items that will Appear in the App**

### **Items Presented at Each App “Ping”**

All items will be prefaced with **“*Right now*….”**

1. How hopeless do you feel? (0 = no hopelessness to 8 = high/severely hopeless)
2. How much self-hate do you feel? (0 = no self-hate to 8 = high/severe self-hate)

- If a score > 3 is endorsed they will also respond to: “How likely do you think this will change in the future?” 0 = never change to 8 = will definitely change

1. How much do you feel like a burden on others? (0 = no burdensomeness to 8 = high/severe burdensomeness)

- If a score > 3 is endorsed they will also respond to: “How likely do you think this will change in the future?” 0 = never change to 8 = will definitely change

1. How agitated (e.g., irritable emotions, like you want to crawl out of your skin) do you feel? (0 = none to 8 = extremely)

- If a score > 3 is endorsed they will also respond to: “How likely do you think this will change in the future? 0 = never change to 8 = will definitely change

1. How connected to others do you feel? (0 = low connection/lonely to 8 = very strong connection)

- If a score < 7 is endorsed they will also respond to: “How likely do you think this will change in the future? 0 = never change to 8 = will definitely change

1. How strong is your urge to self-injure without wanting to die? (slide bar ranging from “none” to “extreme”
2. How strong is your urge to kill yourself? (slide bar ranging from “none” to “extremely strong”)
3. Since the last assessment, did you attempt to kill yourself? 0 = No; 1 = began, but stopped or was interrupted; 2 = Yes, did not need medical attention; 3 = Yes, received medical attention
4. Since the last assessment, did you self-injure without wanting to die (e.g., cutting, burning)? 0 = No; 1 = once; 2 = twice; 3 = 3 or more times
5. To what extent are you stressed by things____:
   - 1. (response range 0 = no stress, to 8 = severely stressed)
   1. at work/school
   2. with a significant other (romantic partner)
   3. in your social life/with friends
   4. at home
   5. financially
   6. related to physical health (e.g., chronic pain, worsening symptoms)
   7. *If a score > 0 is endorsed they will also respond to: “How hopeful are you that this/these stressor(s)
   8. will change in the future? 0 = never change/hopeless to 8= very hopeful/will definitely change
6. To what extent do “see a way out” of your current stressors/situation? 0 = Not at All to 8 = Definitely see a way out

### **Additional items presented only on the first assessment each day**

1. How severe were your sleep problems last night (e.g., trouble falling or staying asleep, waking up too early)? 0 = None/no difficulties to 8 = Very Severe problems/difficulties

2. How many hours did you sleep last night? (sliding bar from 0 – 12 or more hours)

### **Additional items presented only on the last (4^th^) assessment each day**

1. Did you take your psychiatric medications as prescribed today? (No, Some, Yes)

2. In the past 24 hours which of the following substances did you use? (mark all that apply)

- tobacco products (cigarettes, chewing tobacco, cigars)
- Alcoholic beverages (beer, wine, liquor, etc.)
- Cannabis (marijuana, pot, grass, hash, etc.)
- Electronic Vaping products (e-cigarettes)
- Methamphetamine (e.g., meth)
- Other drugs
- None

3. Which of the following is accurate about your substance use in the past 24 hours?

No change in frequency (how often) and amount (quantity)

- Increased frequency (how often) and
- increased amount (quantity)
- Decreased frequency (how often) and decreased amount (quantity)
- Increased frequency (how often) but the decreased amount (quantity)
- Decreased frequency (how often) but increased amount (quantity)

Below is a sample screenshot of what different items types would look like on participants’ phones: We cannot provide actual screenshots of what the above items will look like until the software is purchased, which can only happen after IRB approval is obtained (to ensure the study will occur).


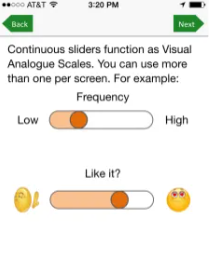

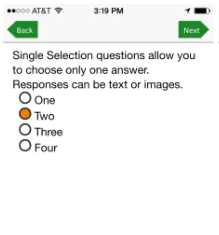


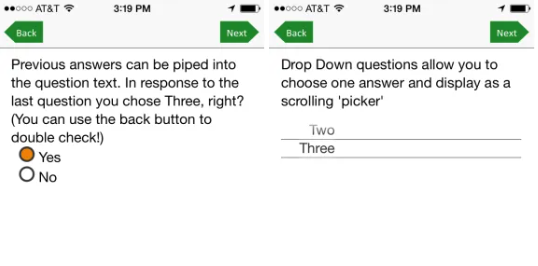

Supplement: Multimedia Appendix 2 [file resprot_v11i7e37583_app2.docx]
